# Effects of Transgenic Maize DBN9936 (*Cry1Ab*+*EPSPS*) and Maize Borer Feeding on Non-Target Organism *Euborellia annulipes* Lucas (Dermaptera: Anisolabididae)

**DOI:** 10.3390/plants14233559

**Published:** 2025-11-21

**Authors:** Zhixiang Fang, Laipan Liu, Wenjing Shen, Li Zhang, Qi Yu, Zhentao Ren, Xin Yin, Biao Liu

**Affiliations:** Key Laboratory on Biodiversity and Biosafety, Nanjing Institute of Environmental Sciences, Ministry of Ecology and Environment, Nanjing 210042, China; zxfang23@126.com (Z.F.); liulaipan@163.com (L.L.); swj@nies.org (W.S.); huanghe70885@sina.com (L.Z.); 15968821722@163.com (Q.Y.); rztkkk@163.com (Z.R.); njfu_shin@163.com (X.Y.)

**Keywords:** transgenic maize, Cry1Ab protein, non-target organisms, *E. annulipes*

## Abstract

The impacts of transgenic maize on non-target organisms serve as a crucial parameter for evaluating the environmental safety of transgenic maize. In the present study, feeding experiments were carried out on *Euborellia annulipes* Lucas (Dermaptera: Anisolabididae) using two types of diets: maize (DBN9936 and parental maize DBN318) and maize combined with maize borers *Ostrinia furnacalis* Guenée (Lepidoptera: Crambidae). The survival rate, body weight, body length, reproductive efficiency, and activities of the superoxide dismutase (SOD) and catalase (CAT) of the predator were monitored. Moreover, the residual concentration of exogenous protein in the bodies, eggs, and feces of the earwigs was measured. The experimental findings indicated that there were no significant disparities in the aforementioned indicators between the transgenic and non-transgenic maize treatment groups. Nevertheless, differences were detected between the maize treatment groups and the maize borer treatment groups, especially regarding egg production. The earwigs in the maize borer treatment groups produced significantly more eggs. Overall, the feeding experiments demonstrated that transgenic maize did not exert adverse effects on *E. annulipes*.

## 1. Introduction

Since the 1990s, the amount of acreage devoted to transgenic maize cultivation has progressively increased. As of 2022, 15 countries cultivated transgenic maize across 66.2 million hectares, representing 32.9% of the global maize cultivation area [1]. Genetically modified crops with insect resistance have been developed to efficiently manage target pests and decrease pesticide application [2]. The commercial introduction of transgenic maize has led to the need to assess the possible impacts of this technology on the environment, especially the effects on non-target organisms [3,4]. Some studies have focused on arthropods at field and laboratory levels [5,6,7], some have examined the effects on soil microorganisms [8,9] and soil-associated meso- and macro-fauna [10]. However, few experiments have been conducted in China on the omnivorous natural enemy of earwigs. Earwig biological testing on transgenic maize is needed before its large-scale commercial cultivation.

*Euborellia annulipes* Lucas (Dermaptera: Anisolabididae) belongs to the *Euborellidae* family within the Dermaptera order and is globally distributed and widespread within the Dermaptera order [11]. They are nocturnal creatures that hide in small caves and crevices or beneath rocks or debris during the day. This highly voracious nocturnal predator consumes prey from various insect orders, including Diptera [12,13], Hemiptera [14], Lepidoptera [15,16], and Coleoptera [17], making it a promising candidate for biological control programs. In China, *E. annulipes* has been detected in more than ten provinces. As omnivorous animals, they prey on diverse prey in various crop fields [18]. Our early investigation revealed that in Chinese maize fields, *E. annulipes* is also a natural enemy that consumes the larvae and eggs of the maize borers *Ostrinia furnacalis* Guenée (Lepidoptera: Crambidae), which aligns with the findings of Calumpang et al. [19]. Additionally, when insect prey is scarce, it feeds on plants, fruits, and even pollen. Owing to its suitability for laboratory rearing, related studies have been conducted abroad [20,21,22], but the research on *E. annulipes* in China remains limited.

Currently, the transgenic maize DBN9936 (*Cry1Ab*+*EPSPS*) has been planted in several provinces in China and will be progressively promoted for widespread cultivation in the future. Some new studies have shown that Cry1Ab protein does not have adverse effects on non-target organisms [23,24]. This study focused on the transgenic maize DBN9936 and its parental maize DBN318 and simulated the feeding behavior of *E. annulipes* under natural conditions. We hope to observe whether transgenic maize will have effects on the *E. annulipes* through direct or indirect feeding methods. Feeding experiments were conducted on diets fed to the earwigs, consisting of two types of maize with or without maize borers (*O. furnacalis*). We monitored the survival rate, weight, length, reproductive efficiency, and activities of the superoxide dismutase (SOD) and catalase (CAT) enzymes of the earwigs. SOD and CAT are widely employed to indicate antioxidant responses underlying the toxic effects of test substances [25]. Furthermore, we determined the residual concentration of the Cry1Ab protein in the earwigs’ bodies, eggs, and feces. On the basis of these data, we aimed to develop a deeper understanding of the impact of transgenic maize on this omnivorous insect prior to its large-scale commercial cultivation in China. 

## 2. Results

### 2.1. The Survival Rate, Body Weight, and Length of the Earwigs

#### 2.1.1. Survival Rate

**Figure 1:**
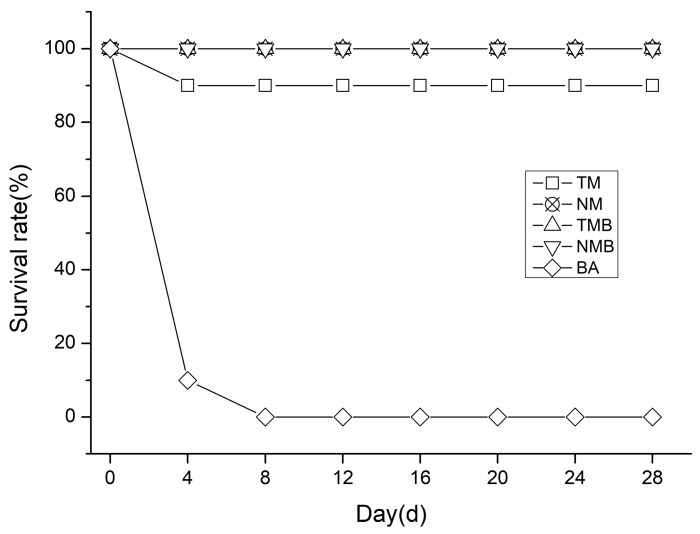
Survival rates of the different treatment groups at 28 days. Transgenic maize treatment (TM); non-transgenic maize treatment (NM); transgenic maize borer treatment (TMB); non-transgenic maize borer treatment (NMB); and Boric Acid treatment (BA).

As shown in Figure 1, in our independent positive control experiment, all earwigs in the BA group died within eight days, confirming their consumption of the artificial diet. In the remaining four treatments, only one earwig in the TM group died during the 28-day experiment, while the other three treatments demonstrated a 100% survival rate (shown in Figure 1 as an over-lapping state). No significant differences were detected between the transgenic and non-transgenic maize treatment groups.

#### 2.1.2. Body Weight

**Figure 2:**
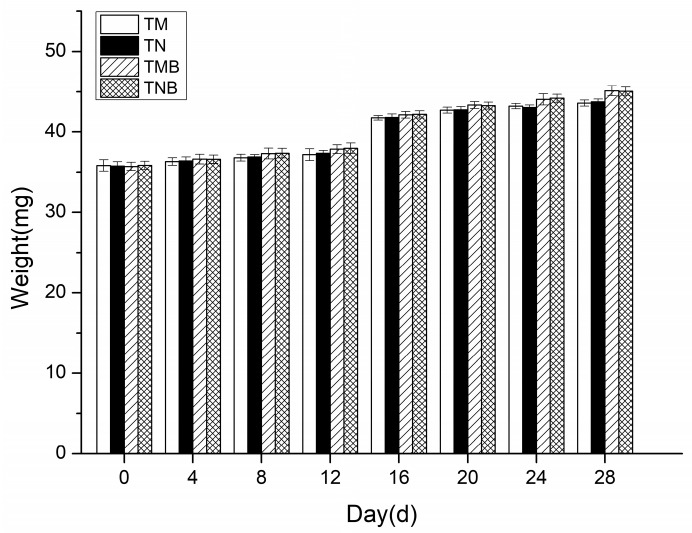
Body weights of earwigs in the four treatment groups. Transgenic maize treatment (TM); non-transgenic maize treatment (NM); transgenic maize borer treatment (TMB); and non-transgenic maize borer treatment (NMB).

The earwigs’ body weight changes over the 28-day experiment are presented in Figure 2. Measurements taken every four days revealed a gradual increase in weight across all the treatments. No significant differences were detected between transgenic and non-transgenic maize in either the artificial diet (*p* > 0.05) or maize borer-supplemented diet (*p* > 0.05). In the 28 days, the earwigs in each treatment fed, moved normally, and gradually increased in weight, with no significant differences between the treatments, indicating that the weight of the earwigs fed the genetically modified maize was not adversely affected.

#### 2.1.3. Body Length

**Figure 3:**
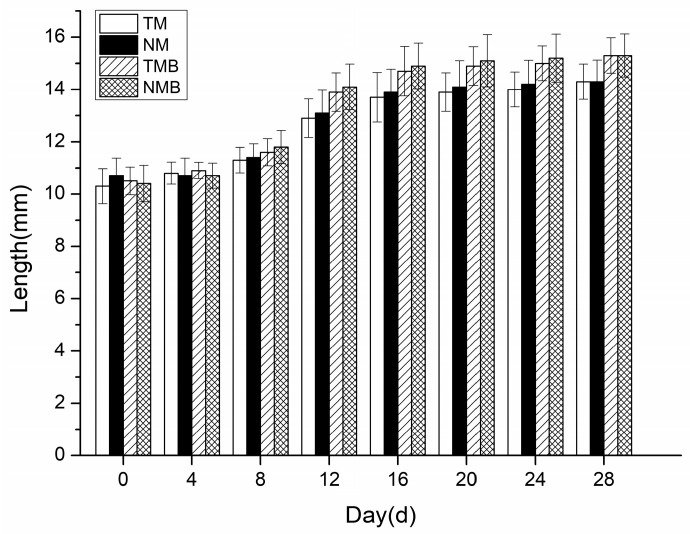
Body length of earwigs in four treatments: transgenic maize treatment (TM), non-transgenic maize treatment (NM), transgenic maize borer treatment (TMB), and non-transgenic maize borer treatment (NMB).

The body length changes in the earwigs over the 28-day experiment are presented in Figure 3. No significant differences were detected between the transgenic and non-transgenic maize in either the artificial diet (*p* > 0.05) or the maize borer-supplemented diet (*p* > 0.05). In the 28 days, the earwigs in each treatment group gradually increased in length, with no significant differences between the treatments, indicating that the length of the earwigs fed the transgenic maize was not adversely affected.

### 2.2. SOD and CAT Enzyme Activity in Earwigs After Survival Experiment

The superoxide dismutase (SOD) activity at the end of the 28-day experiment is summarized in Table 1. No significant differences were found between the TM treatment and the NM treatment. Both treatments resulted in greater SOD activity than the TM or NM treatment, and the difference was significant (*p* ≤ 0.05).

The catalase (CAT) activity was slightly greater in the TMB and NMB treatment groups than in the TM and NM treatment groups, although the difference was not significant (*p* > 0.05). No notable differences were observed between the transgenic and non-transgenic maize treatment groups.

### 2.3. Reproduction of the Earwigs

The egg production across treatment groups is detailed in Table 2. During spawning, the earwigs fed maize borer larvae produced significantly more eggs than those in the pure maize treatment group, but no differences were detected between transgenic and non-transgenic maize groups.

After artificial incubation, the emergence was marginally greater in the maize borer treatment groups (TMB and NMB treatments) than in the pure artificial diet treatment groups (TM and NM treatments), although the difference was not significant (*p* > 0.05). Overall, no significant differences in emergence were detected between the treatment groups.

### 2.4. Cry1Ab Protein Concentrations in Earwigs

After the reproduction experiment, we detected the Cry1Ab protein in earwigs’ tissues and eggs. No Cry1Ab protein was detected in earwig tissues or eggs across all the treatment groups, while the Cry1Ab protein from the transgenic maize DBN9936 was detected in the feces of earwigs in the TM treatment (transgenic maize) and TMB treatment (transgenic maize + borer) groups (Table 3).

## 3. Discussion

*E. annulipes* is an omnivorous insect in agricultural fields [11]. In this study, *E. annulipes* was employed as a non-target organism to assess the biological safety of transgenic maize on omnivorous insects. To simulate natural earwig conditions, two feeding methods were established: (1) a maize-based artificial diet and (2) a maize-based artificial diet supplemented with maize borer larvae.

### 3.1. Survival Experiment

During the 28-day rearing period, the earwigs in every treatment group exhibited normal growth, except for those exposed to Boric Acid, which resulted in mortality within 8 days. These findings demonstrate that both feeding methods effectively evaluated the environmental safety of transgenic maize on *E. annulipes*, confirming that transgenic maize did not cause earwig mortality. These results align with previous research by Cocco et al. [21] and da Silva et al. [22]. Additionally, the earwigs’ body weight and length were monitored throughout the experiment. Our statistical analysis revealed no significant differences in these parameters between the transgenic and non-transgenic maize treatment groups, which is consistent with the findings of Frizzas et al. [26]. Notably, earwigs that were fed diets containing maize borer larvae demonstrated marginally greater body weights and lengths than those fed pure maize diets, although this difference was not statistically significant. Survival experiments confirmed that DBN9936 and its parental maize DBN318 had no effects on the earwigs’ mortality, body weight, or body length. However, earwigs in the insect-supplemented treatment group demonstrated improved growth.

Following the survival experiment, superoxide dismutase (SOD) and catalase (CAT) activities were measured in each treatment, as these two enzymes are often used in toxicology experiments. No significant differences in these enzymes were observed between the transgenic and non-transgenic maize groups, which is consistent with the findings reported by Bai et al. [27] on *Folsomia candida* Willem (Entomobryomorpha: Isotomidae). However, earwigs that were fed maize borer larvae demonstrated elevated activities of both enzymes, with a statistically significant increase in SOD activity. These findings suggest that incorporating maize borer larvae into the diet may trigger physiological responses in omnivorous insects, leading to an increased activity of certain essential enzymes.

### 3.2. Reproduction Experiment

The reproduction experiment revealed no significant differences in egg production or hatching rates between the transgenic and non-transgenic maize treatment groups. However, compared with earwigs that were fed maize borer larvae, those fed maize-only diets demonstrated significantly reduced egg production. Rankin et al. [28] reported similar trends, reporting greater reproductive efficiency in female earwigs fed protein-rich diets (e.g., cat food) than in those maintained on honey and fructose. These findings, including our results, suggest that predation on other insects enhances earwig growth and reproduction.

Finally, the Cry1Ab protein content was analyzed in earwig tissues, eggs, and feces. No Cry1Ab protein was detected in earwig tissues or eggs across any treatment groups. Cry1Ab protein was detected only in the fecal matter from the transgenic maize treatments, indicating that part of the diet is not digested, and some Cry1Ab protein passed through the digestive tract into the excrement. In this experiment, the maize powder added in the artificial feed is produced by freeze-drying and crushing fresh leaves. A study reported that the concentration of *Cry1Ab* in leaves of maize DBN9936 was from 31.20 to 88.27 μg/g [29]. The residual protein detected in the feces of *E. annulipes* in this study might come from undigested plant residues.

## 4. Materials and Methods

### 4.1. Experimental Animals

*E. annulipes* was sourced from non-transgenic maize fields in Yunnan Province, where there is no history of planting transgenic maize, and then reared at the Key Laboratory of Environmental Protection Biodiversity and Biosafety in Jiangsu Province, China. Species identification was confirmed on the basis of body and leg colors, as described by Kocarek [30]. Males and females can be distinguished from each other by the pincers at the end of their abdomen.

The maize borer *O. furnacalis* was sourced from non-transgenic maize fields in Yunnan Province, where there is no history of planting transgenic maize, and reared at the Key Laboratory of Environmental Protection Biodiversity and Biosafety in Jiangsu Province, China.

### 4.2. Artificial Diet

The diet mixture consisted of 300 g of ground transgenic maize powder (DBN9936) or its parental maize powder (DBN318) from Beijing Dabeinong Biotechnology Co., Ltd. (Haidian District, Beijing, China), which was created by freeze-drying fresh maize leaves and crushing them into a powder, 50 g of yeast powder, and 1000 mL of water. The mixture was stirred and sterilized and then stored in a refrigerator for future use. When the experiment began, the mixture was placed on a small piece of filter paper for the testers to eat, and we replaced it every day.

### 4.3. Artificial Soil

The artificial soil was composed of 10% peat soil, 20% kaolin, 69% yellow sand, and 1% calcium carbonate. The pH was adjusted to 5.0–7.0, with a water content of approximately 50%, according to ISO 15952 [31]. The soil was then sterilized and dried for future use.

### 4.4. Rearing Conditions

Referring to the experimental method of da Silva [22], earwigs were reared in glass beakers (12 cm diameter × 15 cm height) with an approximately 3 cm layer of artificial soil. The light–dark cycle was set to 12:12 (light–dark), with the light intensity ranging from 20 to 40 lx. The soil moisture was maintained to keep the middle layer slightly moist. The air temperature was maintained at 25 ± 2 °C, with a humidity of 70 ± 10%.

### 4.5. Survival Experiments

Fourth-instar earwigs with similar body lengths and weights were reared in glass beakers. We used 10 glass beakers, with one individual each in every treatment below.

Transgenic maize treatment (TM): Earwigs were fed an artificial diet containing transgenic maize powder (DBN9936). The feed was placed on a small piece of filter paper in the container and replaced daily.

Non-transgenic maize treatment (NM): Non-transgenic maize leaf powder was used instead of the transgenic maize leaf powder, while the other components were the same.

Transgenic maize borer treatment (TMB): Newly hatched maize borer larvae were added to the TM treatment for 24 h, after which the earwigs were placed in the container.

Non-transgenic maize borer (NMB) treatment: Non-transgenic maize leaf powder was used instead of the transgenic maize leaf powder, while the other components were the same.

Boric Acid treatment (BA): This positive control treatment was conducted separately. Boric Acid as positive control was added to the artificial diet to make its mass fraction 2% [32].

All the treatments lasted for 28 days, and the weight, length, and survival of the earwigs were recorded every 4 days.

### 4.6. Reproductive Experiment

Fourth-instar earwigs were fed according to the survival experimental treatments. Each treatment consisted of five replicates, with one male and one female in each replicate. Each container included wet, absorbent paper folded into a W shape to provide a site for females until the first oviposition and hatching. After the first spawning, the number of eggs and hatching rates were recorded.

### 4.7. Enzyme Activity Experiment

At the end of the survival experiment, we sacrificed the earwigs and obtained tissues for subsequent detection. We used the PBS extracting solution with liquid nitrogen to grind and crush the tissue, fully releasing and dissolving enzymes. The superoxide dismutase (SOD) and catalase (CAT) activity in the earwigs was measured using kits from the Nanjing Jiancheng Bioengineering Institute, Nanjing, China, following the manufacturer’s instructions.

### 4.8. Cry1Ab Protein Residue Experiment

After the reproductive experiment, we sacrificed the earwigs, cleaned them after dissection, and obtained tissues for subsequent detection. We carefully collected small green particles of feces from earwigs every day, which exhibited more regular patterns different from the diet. After breeding, we collected eggs for subsequent experiments. Cry1Ab protein levels in individuals from each treatment group were measured using the ELISA with an Envirologix monitoring kit (Portland, ME, USA) following the manufacturer’s instructions.

### 4.9. Statistical Analysis

Data processing and analysis were performed using SPSS 26.0. One-way analysis of variance (ANOVA) and the least significant difference (LSD, α = 0.05) test were used to evaluate differences among treatments.

## 5. Conclusions

This study simulated two pathways by which *E. annulipes* might acquire exogenous proteins from transgenic maize under field conditions. The results demonstrated that the consumption of transgenic maize had no adverse effects on the earwigs’ survival, growth, or reproductive capacity, and Cry1ab protein did not affect earwigs through maize borers. The spread of Cry1ab protein in the food chain might only depend on undigested plant residues. Furthermore, *E. annulipes*, an omnivorous predator, maintained normal survival and reproduction when fed a maize-based diet, whereas the supplementation with maize borer larvae improved growth and reproductive performances. The underlying mechanisms for these occurrences remain unclear, and whether these traits are common among omnivorous insects warrants further investigation.

## Figures and Tables

**Table 1:** SOD and CAT enzyme activities in earwigs after survival experiments.

Diet	Treatment	SOD (U/mg)	CAT (K/mg)
Maize	TM	36.68 ± 0.75 b	0.73 ± 0.05 a
	NM	36.66 ± 0.59 b	0.74 ± 0.04 a
Maize + Maize Borer	TMB	37.81 ± 0.46 a	0.81 ± 0.03 a
	NMB	37.66 ± 0.33 a	0.79 ± 0.03 a

Different letters in the same row indicate significant differences (*p* ≤ 0.05).

**Table 2:** Egg number and emergence under different treatments.

Diet	Treatment	Egg Number	Emergence (%)
Maize	TM	53.4 ± 5.78 b	73.88 ± 5.34 a
	NM	51.6 ± 6.02 b	74.68 ± 3.99 a
Maize + Maize Borer	TMB	64.6 ± 5.04 a	77.65 ± 2.95 a
	NMB	62.4 ± 4.08 a	76.26 ± 4.75 a

Different letters in the same row indicate significant differences (*p* ≤ 0.05).

**Table 3:** Concentrations of the Cry1Ab protein in the organs, eggs, and feces.

Diet	Treatment	Tissues (ng/g)	Eggs (ng/g)	Feces (ng/g)
Maize	TM	-	-	383.6 ± 15.42 a
	NM	-	-	-
Maize + Maize Borer	TMB	-	-	364.8 ± 12.32 a
	NMB	-	-	-

Different letters in the same row indicate significant differences (*p* ≤ 0.05).

## Data Availability

The original contributions presented in this study are included in the article; further inquiries can be directed to the corresponding author.
